# Frequency and Types of Anemia in Primary Hypothyroidism Patients: A Prospective Observational Study

**DOI:** 10.7759/cureus.59350

**Published:** 2024-04-30

**Authors:** Syed Shahiq Ali, Muhammad Noman Khan, Fatima Zafar, Syed Tariq Ali Adnan, Anusha Yusuf, Muhammad Hussnain, Adnan Anwar, Atif A Hashmi

**Affiliations:** 1 Internal Medicine, Jinnah Postgraduate Medical Centre, Karachi, PAK; 2 Emergency Medicine, Usman Memorial Hospital, Karachi, PAK; 3 Obstetrics and Gynecology, Abbasi Shaheed Hospital, Karachi, PAK; 4 Community Medicine, Karachi Medical and Dental College, Karachi, PAK; 5 Emergency Medicine, SHED Hospital, Karachi, PAK; 6 Internal Medicine, Hamdard College of Medicine and Dentistry, Karachi, PAK; 7 Physiology, Hamdard College of Medicine and Dentistry, Karachi, PAK; 8 Internal Medicine, Essa General Hospital, Karachi, PAK; 9 Pathology, Liaquat National Hospital and Medical College, Karachi, PAK

**Keywords:** normocytic anemia, microcytic anemia, macrocytic anemia, hypothyroidism, anemia

## Abstract

Background

Primary hypothyroidism is a common endocrine disorder resulting from inadequate production of thyroid hormones. Anemia is a common condition that can occur in hypothyroidism. Anemia may occur due to nutrient deficiency, such as iron or vitamin B12 deficiency due to chronic disease in hypothyroidism. Therefore, it is important to evaluate the cause of anemia in hypothyroidism.

Objective

The aim of this study was to determine the frequency of anemia and its types in patients with primary hypothyroidism.

Methods

This was a prospective cross-sectional observational study conducted at the Department of Medicine, Jinnah Postgraduate Medical Center, Karachi, Pakistan, using non-probability consecutive sampling. A total of 176 adults aged 18-65 years of either gender, newly diagnosed with primary hypothyroidism, or with any of its symptoms were included in the study. Patients already on anti-thyroid medication and with post-thyroidectomy hypothyroidism were excluded from the study. The duration of the study was 1.5 years, from January 2020 to July 2021. After ethical approval, written informed consent was obtained from each patient. Demographical data along with results of complete blood picture, including Hb and MCV for diagnosing anemia and its types were recorded on a pre-designed proforma. The chi-square test was applied keeping p < 0.05 as statistically significant.

Results

The mean age of the patients was 42.19 ± 8.43 years, with 59.66% (n = 105) females and 40.34% (n = 71) males. A total of 67% (n =118) patients were found to be anemic. Of these, 38.64% (n = 68) patients had normocytic anemia, 19.32% (n = 34) microcytic anemia, and 9.25% (n = 16) patients had macrocytic anemia; 56.34% (n = 40) males and 74.29% (n = 78) females were reported to be anemic (p = 0.01).

Conclusion

In our study, the frequency of anemia in patients with hypothyroidism was high, with normocytic anemia being the most common type. It is important to know the type of anemia in hypothyroidism, as normocytic anemia is due to the chronic disease process (anemia of chronic disease) and may not respond to nutrient supplementation. Conversely, microcytic anemia is commonly due to iron deficiency and macrocytic anemia is due to vitamin B12 deficiency and therefore, they require replacement therapy. In any case, it is important to identify and treat the underlying cause of anemia.

## Introduction

Anemia represents a significant health concern worldwide, affecting individuals of all ages and demographics [1]. Primary hypothyroidism, a common endocrine disorder resulting from inadequate production of thyroid hormones, is a potential contributor to the development of anemia [2]. Understanding the frequency and types of anemia in patients with primary hypothyroidism is essential for optimizing patient care and improving clinical outcomes [3].

Primary hypothyroidism arises from various etiological factors, including autoimmune thyroiditis (Hashimoto's thyroiditis), iodine deficiency, thyroid surgery, medications, and radiation therapy [4]. The condition is associated with a myriad of systemic manifestations, ranging from fatigue, weight gain, and cold intolerance to cardiovascular, neurological, and hematological abnormalities. Among these, hematological abnormalities, particularly anemia, have garnered significant attention because of their potential impact on patient morbidity and quality of life [5].

The prevalence of anemia in patients with primary hypothyroidism varies across studies and is influenced by multiple factors, including patient demographics, disease severity, and comorbidities [6]. Research indicates that anemia is a common comorbidity in primary hypothyroidism, with reported prevalence rates ranging from 30% to 60% in affected individuals. This elevated prevalence underscores the importance of recognizing and addressing anemia as a potential complication of primary hypothyroidism [7].

Several types of anemia have been documented in primary hypothyroidism patients, each with distinct underlying pathophysiological mechanisms: Normocytic normochromic anemia, characterized by normal-sized red blood cells with normal hemoglobin content, is one of the most prevalent types of anemia observed in primary hypothyroidism [8]. The underlying pathophysiology involves impaired erythropoiesis due to thyroid hormone deficiency, leading to decreased red blood cell production despite adequate iron stores and erythropoietin levels [9]. Macrocytic anemia, characterized by larger-than-normal red blood cells, is another common type of anemia associated with primary hypothyroidism. Thyroid hormone deficiency affects the deoxyribonucleic acid (DNA) synthesis and maturation of red blood cells, resulting in ineffective erythropoiesis and macrocytic erythrocyte production [10]. Iron deficiency anemia (IDA) may coexist with primary hypothyroidism due to impaired iron metabolism. Thyroid hormones play crucial roles in iron absorption, transport, and utilization. Deficiency of thyroid hormones can disrupt these processes, leading to decreased iron availability for erythropoiesis and the development of IDA [11]. Megaloblastic anemia, characterized by enlarged erythrocytes and hyper-segmented neutrophils, can occur in patients with primary hypothyroidism, particularly in those with concurrent pernicious anemia or vitamin B12 deficiency. Thyroid hormone deficiency can intensify the underlying vitamin B12 deficiency by impairing its absorption and utilization, further contributing to megaloblastic erythropoiesis [12]. Autoimmune hemolytic anemia (AIHA), characterized by the destruction of erythrocytes by autoantibodies, is associated with autoimmune thyroid disorders, including primary hypothyroidism. The exact pathogenesis underlying the association between AIHA and primary hypothyroidism remains to be fully elucidated [13]. In conclusion, anemia is a common hematological manifestation observed in patients with primary hypothyroidism, with various types of anemia documented in this population. Normocytic normochromic anemia and macrocytic anemia are among the most prevalent types, reflecting impaired erythropoiesis and altered red blood cell morphology associated with thyroid hormone deficiency [14]. In addition, iron deficiency anemia, macrocytic anemia, and autoimmune hemolytic anemia may also occur in patients with primary hypothyroidism, highlighting the multifactorial nature of anemia in this context. Understanding the frequency and types of anemia in patients with primary hypothyroidism is essential for appropriate diagnostic evaluation and targeted management strategies to optimize patient outcomes [15].

The objective of this study was to determine the frequency and type of anemia in patients with primary hypothyroidism.

## Materials and methods

This cross-sectional observational study was conducted using a non-probability consecutive sampling technique at the Department of Medicine, Jinnah Postgraduate Medical Center, Karachi. The duration of the study was approximately one year and six months, from January 1st, 2020, to June 30th, 2021. Ethical approval was obtained from the Institutional Review Board of Jinnah Postgraduate Medical Center (reference No. F.2-81/2019-GENL/23517/JPMC.

Sample size calculation

The sample size was calculated using the following formula:

N = Z2x (P (100-P)/d2

where P = was the expected frequency of anemia in patients with primary hypothyroidism at 33.77%, Z = 1.96, and d = 7.0% (16).

All adult patients aged 18-65 years of either gender with newly diagnosed primary hypothyroidism were included in the study. These cases were diagnosed, based on high serum TSH and low serum T3 and T4. Cases with TSH >4.2ulU/ml with low serum T3/T3 with clinical evidence of hypothyroidism were labeled as primary hypothyroid patients. Cases of secondary hypothyroidism were excluded. Patients on any antithyroid medication such as amiodarone, carbimazole, propylthiouracil, and radioiodine were excluded from the study. Post-thyroidectomy hypothyroid patients were also excluded from the study.

After the approval of the research proposal, 176 patients with primary hypothyroidism in the Department of Medicine JPMC were included. Written and informed consent was obtained from each patient before inclusion in the study. Data collection included patient demographical and baseline variables like age, BMI, gender, and smoking history. After inclusion in the research, 5 ml venous blood samples were collected from each patient and sent for a complete blood count, including MCV. Anemia was diagnosed based on low Hb (<13.5g/dl in males and <11.5g/dl in females). Further categorization was based on MCV. An MCV <76fl was taken as microcytic, 76-96fl was taken as normocytic and >96fl was categorized as macrocytic anemia. All data were recorded using a predesigned proforma.

Statistical analysis

Data were entered and analyzed using IBM SPSS Statistics for Windows, Version 26 (Released 2019; IBM Corp., Armonk, New York, United States). For quantitative variables such as age, weight, height, body mass index (BMI), and duration of symptoms, the mean and standard deviation were reported. For qualitative variables, categorical variables like gender, history of smoking, presence or absence of anemia, and type of anemia are present were recorded. Stratification of data for effect modifiers such as age, BMI, gender, and duration of symptoms was performed, and the chi-square test was applied keeping p<0.05 as statistically significant to determine the association of effect modifiers with frequency and type of anemia.

## Results

From the total of 176 patients included in the study, the mean age of patients was 42.19 ± 8.43 years, with 54.55% (n = 96) between 18 and 40 years and 45.45% (n = 80) between 41 and 65 years of age. 40.34% (n = 71) patients were male and 59.66 % (n = 105) were female. The mean BMI of patients included in the study was 27.47 ± 2.98 kg/m^2^, with 51.14%(n = 90) of patients below 27 kg/m^2^ and 48.86% (n = 86) above 27 kg/m^2^. The mean duration of primary hypothyroidism was 5.82 ± 2.31 years, with 59.09% (n = 104) patients having the disease for more than five years and 40.91% (n = 72) patients having hypothyroidism for less than five years (Table 1).

**Table 1:** Baseline demographics of patients included in the study (n=176) The data has been presented as n, %.

Variables		n	%
Age (years)	18-40	96	54.55
	41-65	80	45.45
Gender	Male	71	40.34
	Female	105	59.66
BMI (kg/m^2^)	<27	90	51.14
	>27	86	48.86
Duration of hypothyroidism	<5 years	104	59.09
	>5 years	72	40.91

**Figure 1:**
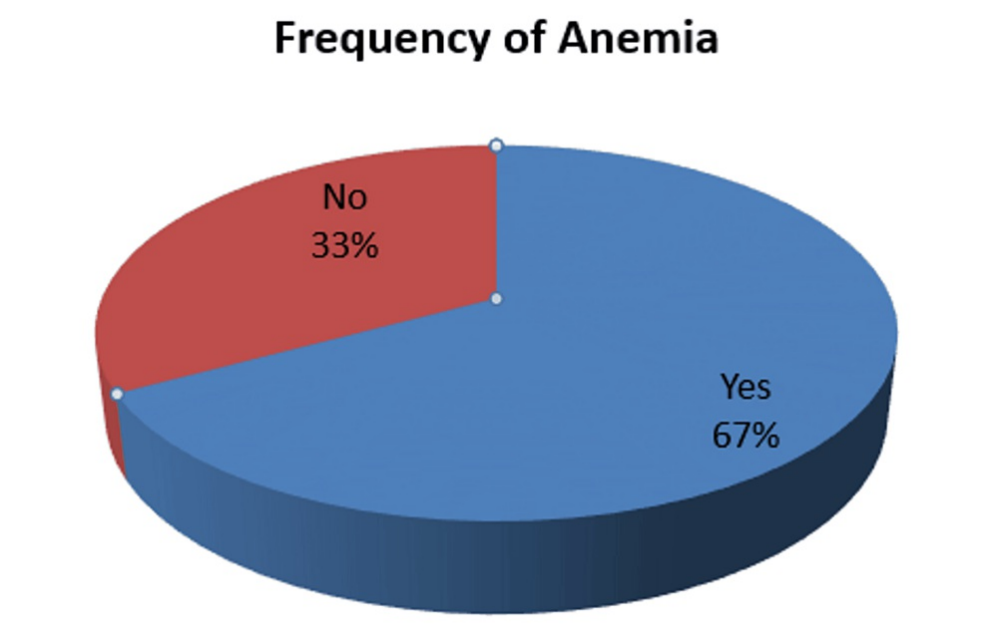
Graphical representation of frequency of anemia in primary hypothyroidism patients (n=176) The data has been presented as %.

Amount the 176 patients, 67% (n = 118) of patients were reported to be anemic, whereas 33% (n = 58) were not found to be anemic (Figure 1).

**Figure 2:**
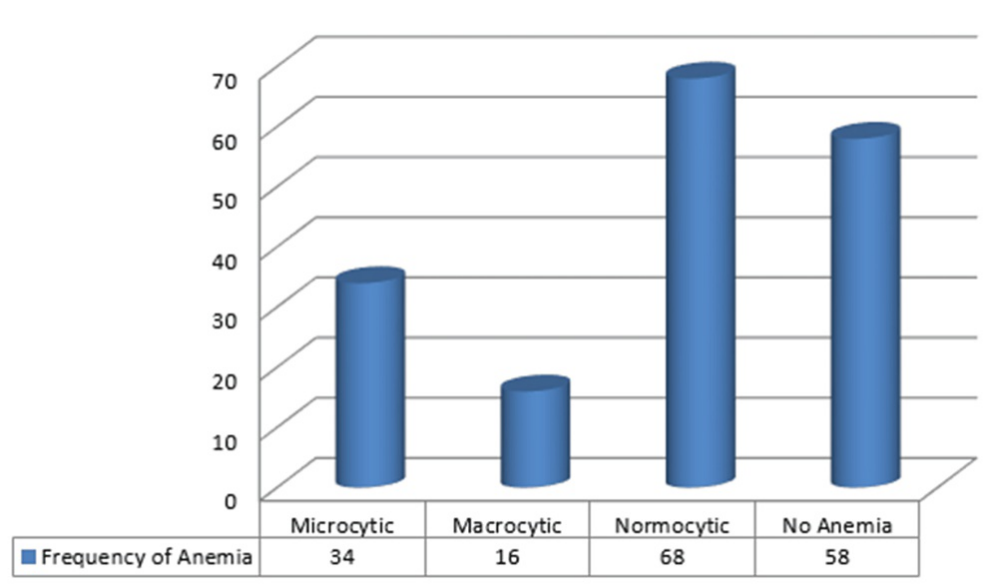
Graphical representation of frequency of types of anemia in primary hypothyroidism patients (n=176) The data has been presented as n.

Figure 2 shows a graphical representation of the frequency of different types of anemia reported in patients. Microcytic hypochromic anemia was observed in 19.32% (n = 34) patients. Macrocytic anemia was observed in 9.25% (n = 16) patients. Normocytic anemia was reported in 38.64% (n = 68) patients, whereas no anemia was observed in 32.95% (n = 58) patients (Figure 2).

In terms of stratification of anemia with demographics, 63.54% (n = 61) of patients with anemia were 18-40 years of age, while 71.25% (n = 57) were approximately 41-65 years of age. An insignificant difference (p = 0.28) was observed between them; 56.34% (n = 40) males and 74.29% (n = 78) females were reported to be anemic, with a significant difference (p = 0.01) between them. In terms of the duration of primary hypothyroidism, 71.15% (n = 74) patients with more than five years of disease were found to be anemic, whereas 61.11% (n = 44) patients with less than five years of primary hypothyroidism were found to be anemic. An insignificant difference (p = 0.16) was observed between them. With regard to BMI, 62.22% (n = 56) of patients with a BMI of 27 kg/m^2^ were reported to be anemic, whereas 72.09% (n = 62) of patients with a BMI greater than 27 kg/m^2^ were found to be anemic. An insignificant difference (p = 0.16) was observed between them, as shown in Table 2.

**Table 2:** Stratification of anemia with demographic variables (n=176) *p-value significant as < 0.05. The data has been presented as n, %. BMI: body mass index.

Variables		Anemia		p-value
		Yes	No	
Age (years)	18-40	61 (63.54 %)	35 (36.46 %)	0.28
	41-65	57 (71.25 %)	23 (28.75 %)	
Gender	Male	40 (56.34 %)	31 (43.66 %)	0.01*
	Female	78 (74.29 %)	27 (25.71 %)	
Duration of disease (years)	<5	74 (71.15 %)	30 (28.85 %)	0.16
	>5	44 (61.11 %)	28 (38.89 %)	
BMI (kg/m^2^)	<27	56 (62.22 %)	34 (37.78 %)	0.16
	>27	62 (72.09 %)	24 (27.91 %)	

## Discussion

The frequency of anemia reported in our study was 67% (n = 118) out of 176 patients with primary hypothyroidism. The highest frequency of anemia type observed in our study was normocytic normochromic in 38.64% (n = 68) patients, followed by microcytic anemia in 19.32 % (n = 34) patients, whereas 9.25% (n = 16) patients were found to be macrocytic anemic. Among stratification of anemia with respect to demographics, only gender was observed to have a significant difference (p = 0.01). A study by Kulkarni et al. [16] reported 75 % (n = 45) of hypothyroidism patients as having anemia, with 65 % (n = 39) reported to have normocytic anemia, followed by microcytic anemia in 23 % (n = 14) of patients and macrocytic anemia in approximately 12 % (n= 7) of patients. Likewise, another study reported normocytic anemia in approximately 46.27 % (n = 902) of patients, 24.36% (n= 475) with microcytic anemia, and 16.36 % (n = 319) with macrocytic anemia. The overall frequency of anemia in primary hypothyroidism reported in this study was 33.77 % (n = 659) [17].

Similar to the findings of our study where primary hypothyroidism was shown to be linked with anemia, El-Masry et al. [18] reported as high as 65 % (n = 39) of adolescent children to be anemic and have hypothyroidism as the primary disease. In yet another study, in hypothyroid patients, the prevalence of anemia was observed to be 43 % (n = 43) in primary (p = 0.0003) and 39 % (n = 39) in subclinical hypothyroidism patients (p = 0.02). The study concluded that an almost equal frequency of anemia was reported in patients with primary/overt subclinical hypothyroidism [19]. Even in congenital hypothyroidism, anemia has been reported as a frequently observed hypothyroidism-associated condition that is dependent on the degree of hypothyroidism [20].

Another study reported 43.3 % (n = 26) microcytic anemia in 60 patients with primary hypothyroidism. The study also recorded no significant difference in terms of vitamin B12, folic acid, and iron levels; therefore, patients with hypothyroidism should be suspected and investigated for anemia [21]. Menorrhagia due to hormonal instability and malabsorption seen in hypothyroidism may result in microcytic anemia. Malabsorption of folic acid and vitamin B12, pernicious anemia, and poor nutrition cause macrocytic anemia. Patients with hypothyroidism are 20 times more likely than the overall population to develop pernicious anemia. Up to 55% of patients with hypothyroidism have macrocytosis, which can arise from thyroid hormone deficiency alone without nutritional deficiency [22].

The overall prevalence of anemia in hypothyroidism ranged from approximately 32% to 67%, with a pooled prevalence of 43.2% (n = 48). The study analyzed data from various observational studies and highlighted the substantial burden of anemia in hypothyroid patients [23].

The prevalence of anemia in patients with hypothyroidism may vary depending on factors such as age, gender, disease severity, and the presence of other comorbidities. Additionally, geographic and ethnic differences may influence the frequency of anemia in hypothyroid patients [24]. For instance, in this study, a significant difference in anemia in hypothyroid patients was observed in terms of gender. However, with age, BMI, and duration of disease, no such significance was recorded.

It should be emphasized that the most common type of anemia found in our cohort of patients with hypothyroidism was normocytic anemia. The most likely cause of microcytic anemia is anemia of chronic disease. Conversely, the common causes of microcytic and macrocytic anemias are iron and vitamin B12 deficiency, respectively [21,22]. Therefore, it is imperative to identify the underlying cause of anemia in hypothyroidism. 

In summary, while estimates may vary, research consistently indicates that anemia is a common comorbidity in hypothyroidism, affecting a significant proportion of patients. Routine screening for anemia should be considered in individuals with hypothyroidism to facilitate early detection and appropriate management, thereby improving patient outcomes and quality of life [25].

Limitations

Our study has a few limitations. First, this was a single-center study, and the sample size was small; therefore, the findings cannot be generalized to the entire population. Moreover, long-term follow-up of the patients was not performed to evaluate the response to iron replacement therapy in patients with iron deficiency anemia. Moreover, further laboratory studies, such as iron studies, thalassemia workup, and vitamin B12 levels were not done to identify the underlying cause of anemia in hypothyroid cases in our study. 

## Conclusions

We found that a substantial number of cases with hypothyroidism in our study cohort of hypothyroid patients were anemic, with normocytic anemia being the most common type. As anemia if left untreated may lead to various complications, including irregular heartbeats and ultimately heart failure, therefore, it is imperative to identify and treat anemia early, especially in hypothyroidism due to underlying symptoms of hypothyroidism that may be complicated by anemia.

In conclusion, we recommend that in patients with hypothyroidism, anemia should be evaluated early. Moreover, because the causes of anemia vary, it is necessary to identify the underlying cause based on the type of anemia. 
